# Effect of Different Amounts of TiF_3_ on the Reversible Hydrogen Storage Properties of 2LiBH_4_–Li_3_AlH_6_ Composite

**DOI:** 10.3389/fchem.2021.693302

**Published:** 2021-05-14

**Authors:** Yun Li, Yuxian Zhang, Lixin Chen

**Affiliations:** ^1^ School of Mechanical and Electrical Engineering, Quzhou College of Technology, Quzhou, China; ^2^ School of Materials Science and Engineering, Zhejiang University, Hangzhou, China

**Keywords:** 2LiBH_4_-Li_3_AlH_6_, hydrogen storage performance, catalytic modification, hydrogen back pressure, reversible performance

## Abstract

Hydrogen is a potential green alternative to conventional energy carriers such as oil and coal. Compared with the storage of hydrogen in gaseous or liquid phases, the chemical storage of hydrogen in solid complex hydrides is safer and more effective. In this study, the complex hydride composite 2LiBH4–Li_3_AlH_6_ with different amounts of TiF_3_ was prepared by simple ball-milling and its hydrogen storage properties were investigated. Temperature programmed desorption and differential scanning calorimetry were used to characterize the de/rehydrogenation performance, and X-ray diffraction and scanning electron microscopy (SEM) were used to explore the phase structure and surface topography of the materials. The dehydrogenation temperature decreased by 48°C in 2LiBH4–Li_3_AlH_6_ with 15 wt% TiF_3_ composites compared to the composite without additives while the reaction kinetics was accelerated by 20%. In addition, the influence of hydrogen back pressure on the 2LiBH4–Li_3_AlH_6_ with 5 wt% TiF_3_ composite was also investigated. The results show that hydrogen back pressure between 2.5 and 3.5 bar can improve the reversible performance of the composite to some extent. With a back pressure of 3.5 bar, the second dehydrogenation capacity increased to 4.6 wt% from the 3.3 wt% in the 2LiBH_4_–Li_3_AlH_6_ composite without hydrogen back pressure. However, the dehydrogenation kinetics was hindered. About 150 h, which is 100 times the time required without back pressure, was needed to release 8.7 wt% of hydrogen at 3.5 bar hydrogen back pressure. The SEM results show that aluminum was aggregated after the second cycle of dehydrogenation at the hydrogen back pressure of 3 bar, resulting in the partial reversibility of the 5 wt% TiF_3_-added 2LiBH4–Li3AlH_6_ composite.

## Introduction

As a new energy source, hydrogen has great potential to solve the serious energy depletion and atmospheric pollution caused by excessive utilization of conventional energy sources. Hydrogen is the most abundant element in the Universe. The calorific value of 1 kg of hydrogen is 140 megajoules of energy, which is three times of that of the amount of energy released by the same weight of oil. Moreover, water vapor is the only combustion product of hydrogen, making it a pollution-free energy source. However, the limitations of hydrogen storage technology have hindered its practical application.

As a promising candidate for solid-state hydrogen storage, LiBH_4_ has a high theoretical gravimetric hydrogen density of 18.5 wt%, which is the highest among all the solid hydride materials. (Züttel et al., 2003) first studied the desorption properties of LiBH_4_. The material released 0.3 wt% hydrogen at 200°C and 1 wt% hydrogen at 320°C. After heating to 500°C, rapid hydrogen desorption was observed, and 9 wt% of hydrogen was released. This capacity meets the requirements of the US Department of Energy (DOE) (Jain et al., 2010), but the desorption temperature is too high for practical applications, and the dehydrogenation kinetics also need to be improved.

Many researchers have focused on the modification of LiBH_4_ using various methods, such as composite modification (Xia et al., 2011; Langmi et al., 2012; Bilen et al., 2015; Liu et al., 2015; Sofianos et al., 2017), catalyst modification (Puszkiel et al., 2014; Ma et al., 2016; Zhang et al., 2018), and nanoengineering (Zhao et al., 2014; Surrey et al., 2016; Sofianos et al., 2018). Compositing LiBH_4_ with other active hydrides is an effective way to enhance its hydrogen storage performance. For example, Liu et al., 2016 milled LiBH_4_ with AlH_3_ to form a 2LiBH_4_ + AlH_3_ composite. The dehydrogenation temperature of the 2LiBH_4_ + AlH_3_ composite was reduced by 30°C compared with pure LiBH_4_, and 11.2 wt% hydrogen was obtained. (Ding et al. 2019) found that in a nano-sized composite of LiBH_4_ and MgH_2_ prepared by ball-milling with aerosol spraying (BMAS), the LiBH_4_ released hydrogen at 265°C, and approximately 5.0 wt% H_2_ was reversible in the first five dehydrogenation and rehydrogenation cycles. Using the shrinking-core model, it was found that a larger interfacial area between LiBH_4_ and MgH_2_ improves the dynamics of the nano 2LiBH_4_ + MgH_2_ mixture (Ding et al., 2019).

Other researchers have prepared binary (Mao et al., 2009b; Yu et al., 2010; Hansen et al., 2013; Li et al., 2013; Mustafa et al., 2018; Yahya et al., 2019) or ternary (Yang et al., 2012; Liu et al., 2021) composite systems of LiBH_4_ and other complex hydrides (Halim Yap et al., 2018; Halim Yap and Ismail, 2018), such as LiBH_4_–LiAlH_4_ (Thaweelap and Utke, 2016; Meethom et al., 2020), LiBH_4_–Li_3_AlH_6_ (Choi et al., 2011b), LiBH_4_–NaBH_4_ (Dematteis et al., 2016), LiBH_4_–NaAlH_4_, LiBH_4_–Mg(BH_4_)_2_ (Zheng et al., 2020), LiBH_4_–Ca(BH_4_)_2_ (Ampoumogli et al., 2015), LiBH_4_–Mg_2_NiH_4_ (Bergemann et al., 2019), LiBH_4_–NaBH_4_–MgH_2_ (Xiang et al., 2018), and LiBH_4_–Li_3_AlH_6_–MgH_2_ (Lin et al., 2020). Bargeman et al*.* (Bergemann, et al. 2019) investigated the reaction mechanism of a LiBH_4_–Mg_2_NiH_4_ composite. They found that the dehydrogenation path was significantly influenced by the back pressure and temperature. When the back pressure was increased to 50 bar, Mg_2_NiH_4_ reacted with solid LiBH_4_ below 270°C, and the reaction enthalpy was reduced by 13 kJ/mol H_2_. In Thaweelap’s work (Thaweelap and Utke, 2016), LiBH_4_ and LiAlH_4_ were synthesized together with Ti-based catalysts or carbon nanotubes to achieve a synergistic effect between the catalysts and composites. Both the kinetics and thermodynamics of the samples were improved. In addition, the reversibility of the LiBH_4_–LiAlH_4_–MWCNT composite was increased to 3.7 wt% in the second cycle compared to 2.8 wt% in the LiBH_4_–LiAlH_4_ composite. Wu (Wu et al. 2012) revealed the reaction mechanism of a LiBH_4_–Li_3_AlH_6_ composite, which received 8.5 wt% hydrogen in the dehydrogenation process. Zhou (Zhou et al. 2017) prepared a 2LiBH_4_–LiAlH_4_ composite nanoconfined in an RFC aerogel, and found that both the thermodynamic and kinetic properties were enhanced.

Some catalysts such as CaH_2_ (Jiang et al., 2012), NbF_5_ (Mao et al., 2013), TiF_3_ (Mao et al., 2010) and NbCl_5_ (Tu et al., 2015) destabilize LiBH_4_ to decompose H_2_ under moderate conditions. Ming mixed LiBH_4_ with several additives using the ball-milling method (Au and Jurgensen, 2006). The modified mixtures desorbed hydrogen at a lower temperature and higher rate. Among the various additives, Ti-based catalysts had an obvious effect. Choi (Choi et al. 2011a) demonstrated that TiCl_3_ could promote desorption in a Li_3_AlH_6_/2LiBH_4_ composite at a lower temperature. Because of the catalyst effect of TiCl_3_, Li_3_AlH_6_ started to release hydrogen at 81°C, and LiBH_4_ released hydrogen at 226°C, which are 130 and 200°C lower than the temperatures at which hydrogen was released by pure Li_3_AlH_6_ and LiBH_4_, respectively. A novel two-dimensional (2D) layered Ti_3_C_2_ material was added to the LiBH_4_–THF solution to synthesize a series of LiBH_4_–χTi_3_C_2_ composites with different mass ratios. The LiBH_4_–2Ti_3_C_2_ composite released hydrogen at 172.6°C, and its activation energy was reduced by 50% compared to that of pure LiBH_4_ (Zang et al., 2018). TiF_3_ is a well known catalyst for solid-state hydrogen storage materials. It effectively improved the hydrogen storage performance of LiAlH_4_–MgH_2_ (Mao et al., 2011), MgH_2_–NaAlH_4_ (Ismail et al., 2012) and 2NaAlH_4_+Ca(BH_4_)_2_ (Mustafa and ismail, 2019) binary composite.

In this study, a ternary composite of 2LiBH_4_–Li_3_AlH_6_ with different amounts of TiF_3_ (the mass percent of TiF_3_ were 5, 10, and, 15%) was prepared by a high-energy ball milling method. The synergistic effects between the composite and the catalyst were investigated together with the influence of the hydrogen back pressure on the reversibility.

## Materials and Methods

LiBH_4_ powder (95% purity) and TiF_3_ powder (99% purity) were purchased from Acros Organics and Sigma Aldrich, respectively. Li_3_AlH_6_ powder was obtained by mixing LiAlH_4_ (95% purity, Sigma Aldrich) and LiH (98% purity, Alfa Aesar) using a high-energy ball-milling method at a ball-to-powder ratio of 40:1 (Li et al., 2020). All the materials used in this work were stored in a glove box purchased from Mikrouna. The glove box was filled with high-purity argon to purge the atmosphere, and the water vapor content was less than 1 ppm. The 2LiBH4–Li3AlH_6_ series with and without TiF_3_ composites was weighed and mixed in this glove box, and then loaded in a sealed stainless ball-milling tank. Planetary ball-milling equipment (QM-3SP4) was used to grind the materials at 300 revolutions per minute for 1 h.

A Sievert-type instrument which recorded the pressure change of the hydrogen released during the heating process was used to evaluate the dehydrogenation/rehydrogenation performance of the materials. Approximately 200 mg of each sample was charged into a thin stainless-steel tube reactor. The reactor was then heated from ambient temperature to the set temperature at a constant heating rate. The time, temperature, and pressure data were recorded every ten seconds. The amount of hydrogen released or absorbed during the heating process was calculated using the ideal gas state equation. Differential scanning calorimetry (DSC, Netzsch STA 449F3/Netzsch Q403C) was used to evaluate the thermal effects of the materials. In the glove box, about 1–1.5 mg of samples were loaded each time and sealed in an aluminum crucible. Both the sample crucible and the reference crucible were placed in the reaction chamber of the DSC equipment, which was filled with flowing high purity argon at a flow rate of 50 ml/min.

X-ray diffraction (XRD, X'Pert-PRO) and scanning electron microscopy (SEM, FEI FSEM SIRION-100) were used to analyze the phase structure and observe the surface topography of the material. The XRD K-Alpha1 and K-Alpha2 wavelengths were 1.540598 Å and 1.544426 Å, respectively. The samples were sealed in a transparent macromolecular container to prevent oxidation. Fourier transform infrared spectroscopy (FT–IR, Bruker, Tensor 27) at the scan rate of 30 cm^−1^/min and resolution of 0.5 cm^−1^ was used for chemical bond analysis. The crucibles used in the experiments were aluminum crucibles.

## Results and Discussion

### DSC Traces of LiBH_4_, Li_3_AlH_6_, and LiBH_4_–Li_3_AlH_6_ Samples

**FIGURE 1:**
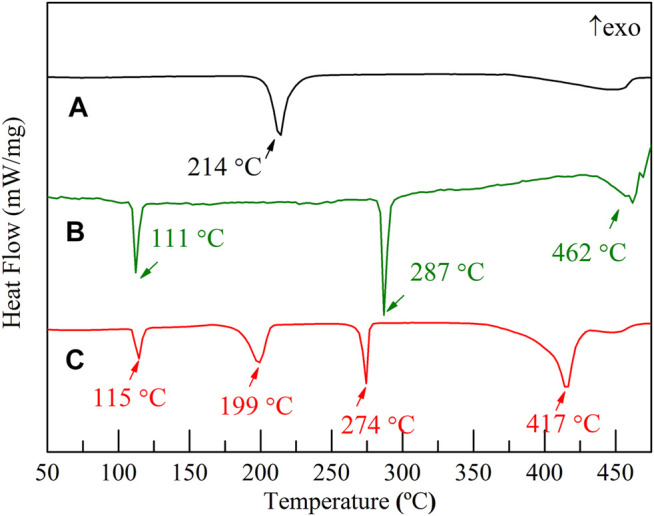
DSC traces of **(A)** Li_3_AlH_6_, **(B)** LiBH_4_, and **(C)** 2LiBH_4_–Li_3_AlH_6_ composite.

The DSC traces of the Li_3_AlH_6_, LiBH_4_, and 2LiBH_4_–Li_3_AlH_6_ samples at the heating rate of 5°C/min from ambient temperature to 500°C are shown in Figure 1. In the Li_3_AlH_6_ sample (Figure 1A), there is an endothermic peak at 214°C due to the decomposition process of Li_3_AlH_6_. The last peak in the DSC trace of Li_3_AlH_6_ was the decomposition of LiH (Aresfernandez, 2007).The decomposition reaction of Li_3_AlH_6_ and the resulting products are described by Eq. 1 (Zang, et al., 2018):

$$Li_{3}\mathrm{A1}H_{6}\to \mathrm{3LiH}+3/2H_{2}$$ (1)

There are three endothermic peaks in the DSC trace of LiBH_4_ shown in Figure 1B. The first peak at 111°C is the lattice transition peak of LiBH_4_, and the second peak at 287°C corresponds to the melting of LiBH_4_ (Züttel, et al., 2003). The third peak is wider than the first two and indicates the decomposition of LiBH_4_ (Li et al., 2017). The decomposition process is shown in Eq. 2 (Mao et al., 2009a):

$$\mathrm{LiB}H_{4}\to \mathrm{LiH}+B+3/2H_{2}$$ (2)

Four endothermal peaks corresponding to the characteristic peaks of the components were observed for the 2LiBH4–Li_3_AlH_6_ composite. The LiBH_4_ in the 2LiBH4–Li3AlH_6_ composite melted at 274°C, which is 13°C lower than the melting point of pure LiBH_4_. The second and the forth endothermal peaks mean a rapid dehydrogenation temperature of Li_3_AlH_6_ and LiBH_4_, were 199 and 417°C (see Figure 1C), which were lower than pure Li_3_AlH_6_ and LiBH_4_, indicating that the dehydrogenation performance of the 2LiBH4–Li_3_AlH_6_ composite obtained a better thermal performance.

### Influence of TiF_3_ on Hydrogen Desorption Performance of 2LiBH_4_–Li_3_AlH_6_ Composite

**FIGURE 2:**
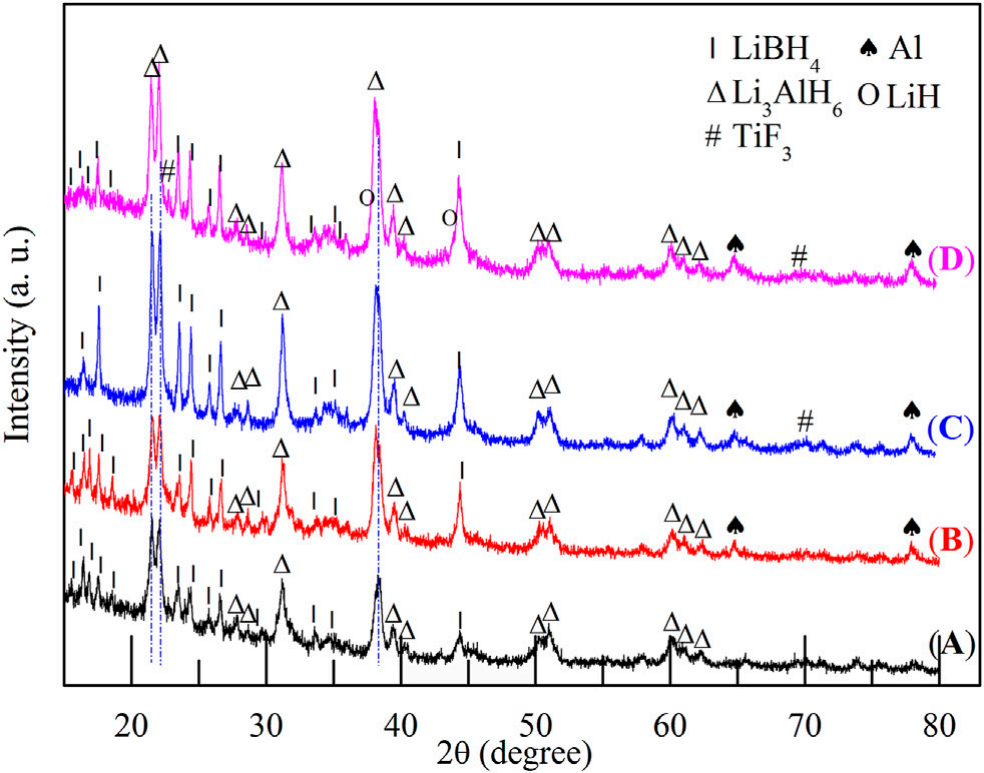
XRD patterns of 2LiBH_4_–Li_3_AlH_6_ samples with different amounts of TiF_3_ after ball-milling: **(A)** 0 wt%, **(B)** 5 wt%, **(C)** 10 wt%, and **(D)** 15 wt%.

**FIGURE 3:**
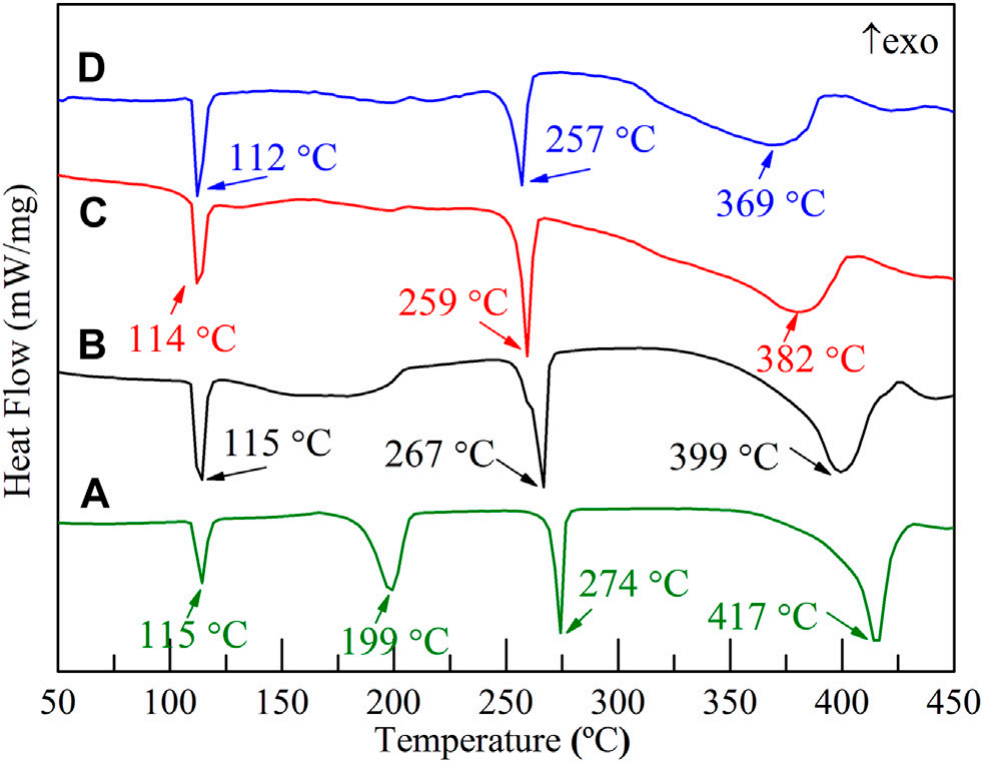
DSC traces of 2LiBH_4_–Li_3_AlH_6_ samples with different amounts of added TiF_3_: **(A)** 0 wt%, **(B)** 5 wt%, **(C)** 10 wt%, and **(D)** 15 wt%.

The hydrogen storage properties of the 2LiBH_4_–Li_3_AlH_6_ composite both in its original state and in the presence of TiF_3_ were investigated in this study. The samples were weighed and loaded in a glove box filled with pure argon. The mass percentages of TiF_3_ in the four samples were 0, 5, 10, and 15 wt%. All the samples were prepared by ball-milling for 1 h, and XRD was used to determine the composition of the samples. LiBH_4_ and Li_3_AlH_6_ peaks were observed in the 2LiBH4–Li_3_AlH_6_ sample without the TiF_3_ additive (Figure 2A). The main diffraction peaks of the three 2LiBH4–Li3AlH_6_ composites with different amount of TiF_3_ (Figures 2B–D) correspond to those of the 2LiBH4–Li3AlH_6_ sample without additive. The weak diffraction peaks at 23° or 70° in Figure 2C and Figure 2D could be attributed to TiF_3_. The absence of a TiF_3_ peak in Figure 2B may possibly be due to the small amount of highly dispersed TiF_3_. As the TiF_3_ content increased, the diffraction peak of Al in the 2LiBH4–Li3AlH_6_ with TiF_3_ composites gradually became obvious, and weak LiH peaks appeared at 38° and 44°. These results indicate that TiF_3_ destabilized Li_3_AlH_6_ by promoting its decomposition into LiH and Al during the ball-milling process (Ares, 2008).Approximately 1 mg of the prepared samples was loaded into an aluminum oxide crucible for the DSC test each time. The results are shown in Figure 3. Comparing the 2LiBH_4_–Li_3_AlH_6_ samples with TiF_3_ (Figures 3B–D) or without TiF_3_ (Figure 3A), the lattice transition peaks of LiBH_4_, which were between 112 and 115°C, were almost unchanged. The melting peak of LiBH_4_ decreased from 274 to 257°C as the amount of TiF_3_ increased. In addition, compared with the 2LiBH4–Li_3_AlH_6_ sample, the Li_3_AlH_6_ decomposition peak shifted obviously to lower temperatures and became wider in the 2LiBH_4_–Li_3_AlH_6_ with 5 wt% TiF_3_. In the samples with 10 and 15 wt% TiF_3_, the typical endothermic peak of Li_3_AlH_6_ was almost undetectable. A possible reason for this is the partial decomposition of Li_3_AlH_6_ during the ball-milling process (Liu, 2010). This result is consistent with the XRD patterns (Figure 2), in which LiH and Al were detected in the TiF_3_-added ball-milling samples, and indicates that TiF_3_ is an effective catalyst that can destabilize Li_3_AlH_6_ at a lower temperature. LiBH_4_ decomposed at 399, 382, and 369°C in the 2LiBH_4_–Li_3_AlH_6_ composite with 5, 10, and 15 wt% TiF_3_ additives, respectively. Faster dehydrogenation kinetics were also obtained as the dehydrogenation peak of LiBH_4_ became broader with the increase in the amount of TiF_3_ (Zang, et al., 2018). The introduction of the TiF_3_ catalyst promoted both the thermal decomposition and the kinetic properties of LiBH_4_ and Li_3_AlH_6_.

**FIGURE 4:**
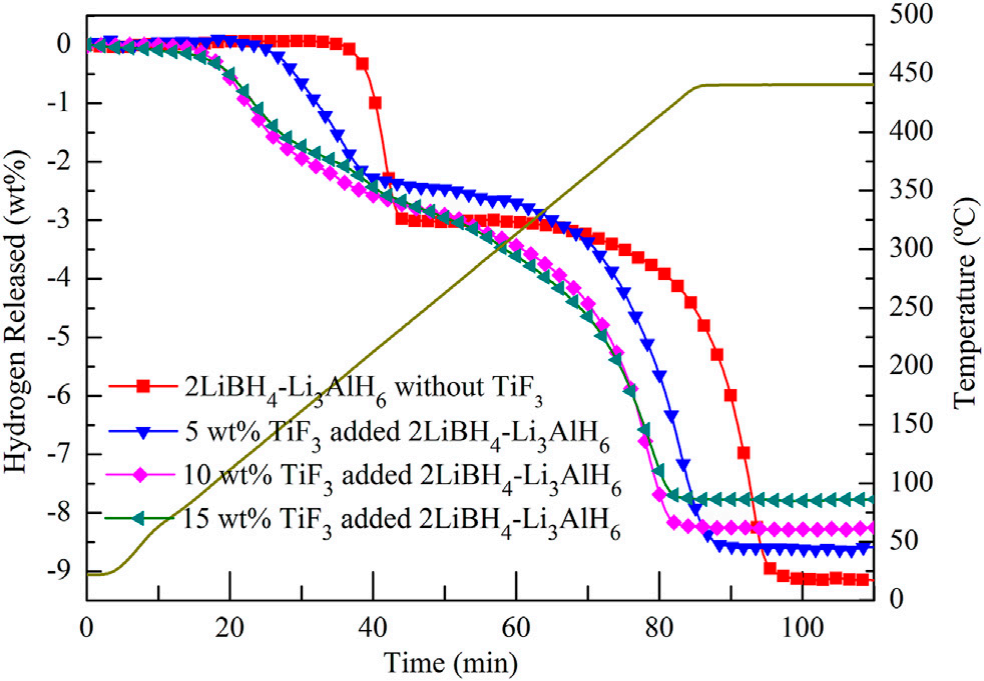
Hydrogen desorption curves of 2LiBH_4_–Li_3_AlH_6_ samples with different amounts of added TiF_3_.

A temperature-programmed desorption (TPD) test was performed to study the dehydrogenation properties with and without TiF_3_ in the 2LiBH_4_–Li_3_AlH_6_ samples. Table 1 shows the theoretical hydrogen storage capacities of the 2LiBH_4_–Li_3_AlH_6_ samples with different amounts of TiF_3_. As the amount of TiF_3_ increased, the weight percentage of H in 1 g of ball-milled sample decreased from 9.24 wt% to 7.85 wt%, as shown in Table 1. Approximately 200 mg of each sample was loaded into a stainless-steel reactor. The reactor was heated from room temperature to 440°C at a heating rate of 5°C/min and maintained at 440°C for half an hour. The hydrogen desorption curves of the four samples are shown in Figure 4. The 2LiBH_4_–Li_3_AlH_6_ sample underwent a two-step dehydrogenation process in which the release of hydrogen began at 190°C and approximately 3 wt% of hydrogen was released in the first step. The second step began at approximately 330 C and the remaining 6.15 wt% of hydrogen was released. The total dehydrogenation capacity of 9.15 wt% is consistent with the theoretical hydrogen storage capacity. In the 2LiBH_4_–Li_3_AlH_6_ with 5 wt% TiF_3_ composite, the initial dehydrogenation temperatures of the two-step process decreased to 110 and 270°C. As the amount of TiF_3_ increased, the decomposition temperatures of Li_3_AlH_6_ and LiBH_4_ tended to decrease. The Li_3_AlH_6_ decomposed at 88°C in the 10 wt% TiF_3_-added 2LiBH_4_–Li_3_AlH_6_ composite, compared to 85°C in the 15 wt% TiF_3_-added sample. LiBH_4_ started to release hydrogen at 238°C in the 10 wt %-TiF_3_ added sample and at 225°C in the 15 wt% TiF_3_ added sample. These results indicate that the addition of TiF_3_ improved the thermodynamic properties of the 2LiBH_4_–Li_3_AlH_6_ composites. In contrast to the 2LiBH_4_–Li_3_AlH_6_ composite, the absence of a “platform area” between the first and second steps of the composites with TiF_3_ indicated that LiBH_4_ tended to decomposition at a lower temperature and the rate of hydrogen desorption were accelerated by the catalytic effect of TiF_3_. The time required for total decomposition decreased to 82 min when the TiF_3_ amount was 15 wt% compared to the 95 min required for the 2LiBH_4_–Li_3_AlH_6_ composite without TiF_3_. Clearly, the addition of TiF_3_ enhanced both the thermodynamic and kinetic properties of the 2LiBH_4_–Li_3_AlH_6_ composite system. However, the theoretical hydrogen storage capacity and the actual hydrogen desorption capacity of the composites with TiF_3_ decreased as the amount of TiF_3_ increased (shown in Table 1).

**TABLE 1:** Theoretical hydrogen storage capacity and actual hydrogen desorption capacity of 2LiBH_4_–Li_3_AlH_6_ samples with different amounts of TiF_3_.

TiF_3_ content (wt%)	0	5	10	15
Theoretical hydrogen storage capacity (wt%)	9.24	8.77	8.31	7.85
Actual hydrogen desorption capacity (wt%)	9.15	8.67	8.27	7.71

**FIGURE 5:**
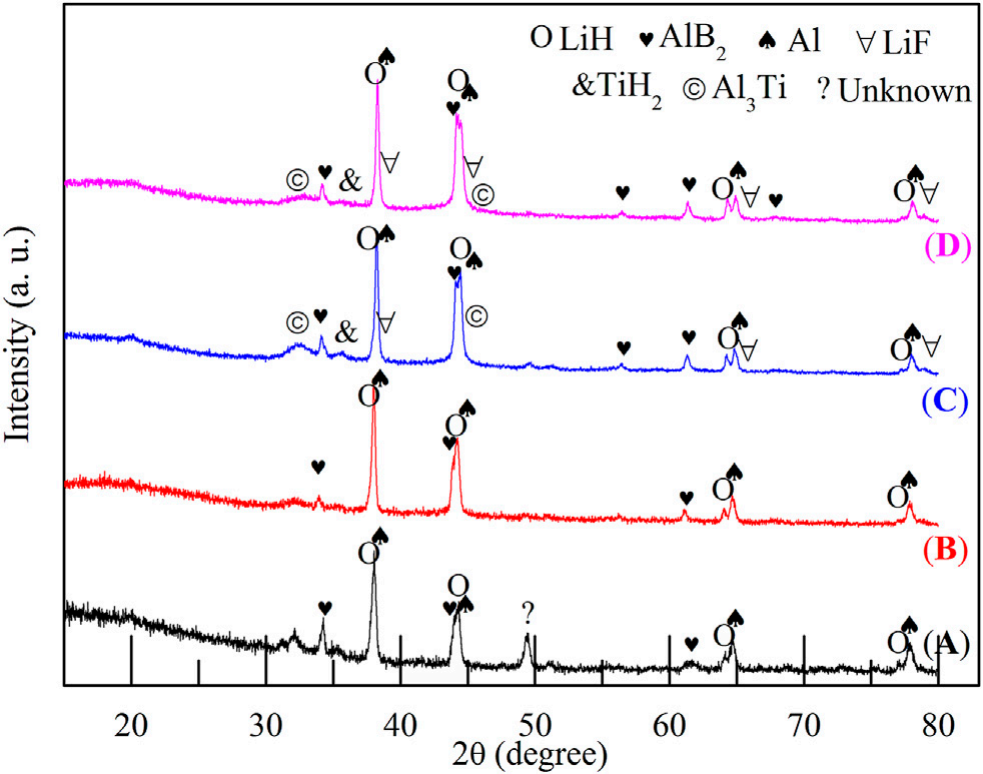
XRD patterns of dehydrogenated 2LiBH_4_–Li_3_AlH_6_ samples with different amounts of added TiF_3_: **(A)** 0 wt%, **(B)** 5 wt%, **(C)** 10 wt%, and **(D)** 15 wt%.

To reveal the dehydrogenation pathway and catalytic effect of TiF_3_, the XRD patterns of the dehydrogenation products of the four samples are displayed in Figure 5. In the 2LiBH_4_–Li_3_AlH_6_ composite (Figure 5A), the presence of LiH, Al, and AlB_2_ phases indicates that LiBH_4_ reacted with Al generated from Li_3_AlH_6_ (Li et al., 2020) *in situ* to form AlB_2_, as shown in Eq. 3 (Choi et al., 2011b; Soru, 2014; Meethom, et al. 2020):

$$\mathrm{2LiB}H_{4}+\mathrm{A1}\to \mathrm{2LiH}+\mathrm{A1}B_{2}+3H_{2}$$ (3)

The unknown peak near 49° was also observed in previous studies (Mao et al., 2009a; Li, 2012). This peak was probably due to an intermediate dehydrogenation product of LiBH_4_ and Li_3_AlH_6_. In the samples with 10 wt% TiF_3_ (Figure 5C) and the 15 wt% TiF_3_ (Figure 5D), some Ti-based or F-based phases could be detected in addition to the peaks corresponding to LiH, Al, and AlB_2_. The LiF characteristic peaks near 38° and 79° correspond to the reaction products between LiBH_4_ and TiF_3_, as shown in Eq. 4 (Mao et al., 2009b; Guo, 2010).

$$\mathrm{LiB}H_{4}+1/3\mathrm{Ti}F_{3}\to \mathrm{LiF}\;+1/3\mathrm{TiB2}+1/3B+2H_{2}\;$$ (4)

The TiH_2_ peaks at 36° and Al_3_Ti peaks at 31° and 44° may possibly be due to reaction products between Li_3_AlH_6_ and TiF_3_, the reaction mechanisms of which were explored in Liu’s work (Liu et al., 2010). The diffraction peak of AlB_2_ appeared at 57° in the 10 and 15 wt% TiF_3_-added samples, indicating that the amount of AlB_2_ was increased.

### Reversible Performance of TiF_3_ Added 2LiBH_4_–Li_3_AlH_6_ Composite with Different Back Pressures

**FIGURE 6:**
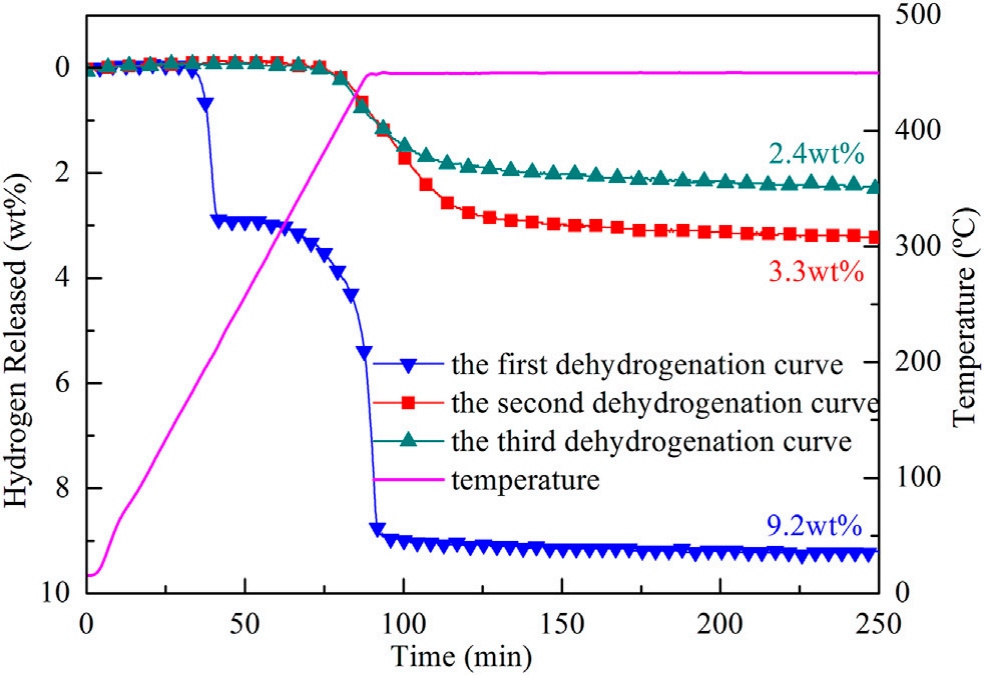
Cyclic dehydrogenation curves of the 2LiBH_4_–Li_3_AlH_6_ composite.

The reversible performance of the 2LiBH_4_–Li_3_AlH_6_ composite was investigated in this study. Approximately 200 mg samples of 2LiBH_4_–Li_3_AlH_6_ composite were loaded in a tubular reactor and heated to 450°C at a heating rate of 5°C/min. The dehydrogenated samples were maintained at 450°C for 250 min and then cooled down to ambient temperature naturally. The hydrogen adsorption process was performed at the hydrogen pressure of 100 bar at 450°C for 10 h. The dehydrogenation curves of the 2LiBH_4_–Li_3_AlH_6_ composite are shown in Figure 6. The hydrogen release capacity of the 2LiBH_4_–Li_3_AlH_6_ composite was 9.2 wt% in the first cycle, and 3.3 wt% and 2.4 wt% in the second and third cycles, respectively. Unlike the two-step desorption reactions of the first cycle, only one-step reactions occurred in the second and third cycles, and the initial dehydrogenation temperature reached as high as 400°C.

**FIGURE 7:**
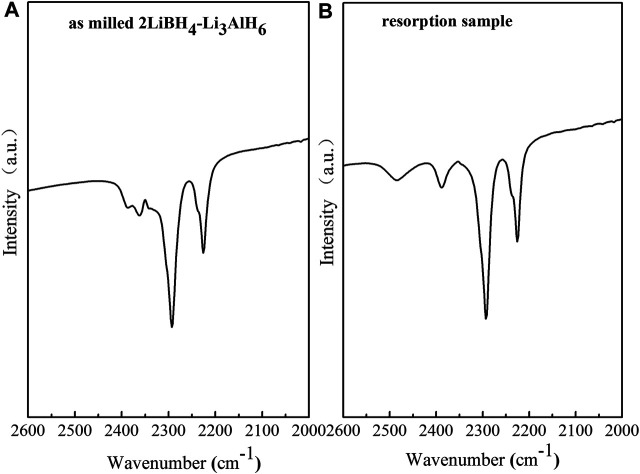
FT–IR curves of the 2LiBH_4_–Li_3_AlH_6_ samples: **(A)** after ball-milling and **(B)** rehydrogenation.

The observation of the B–H bond of LiBH_4_ at 2,200–2,400 cm^−1^ in the FT–IR curves of the ball-milled sample and the resorption sample (Figure 7) indicates that the 2LiBH_4_–Li_3_AlH_6_ composite was partially reversible, and that the reversible component was LiBH_4_. The formation process of LiBH_4_ is given by Eq. 5 (Li et al., 2012):

$$\mathrm{2LiH}+\mathrm{A1}B_{2}+3H_{2}\to \mathrm{2LiB}H_{4}+\mathrm{A1}.$$ (5)

**FIGURE 8:**
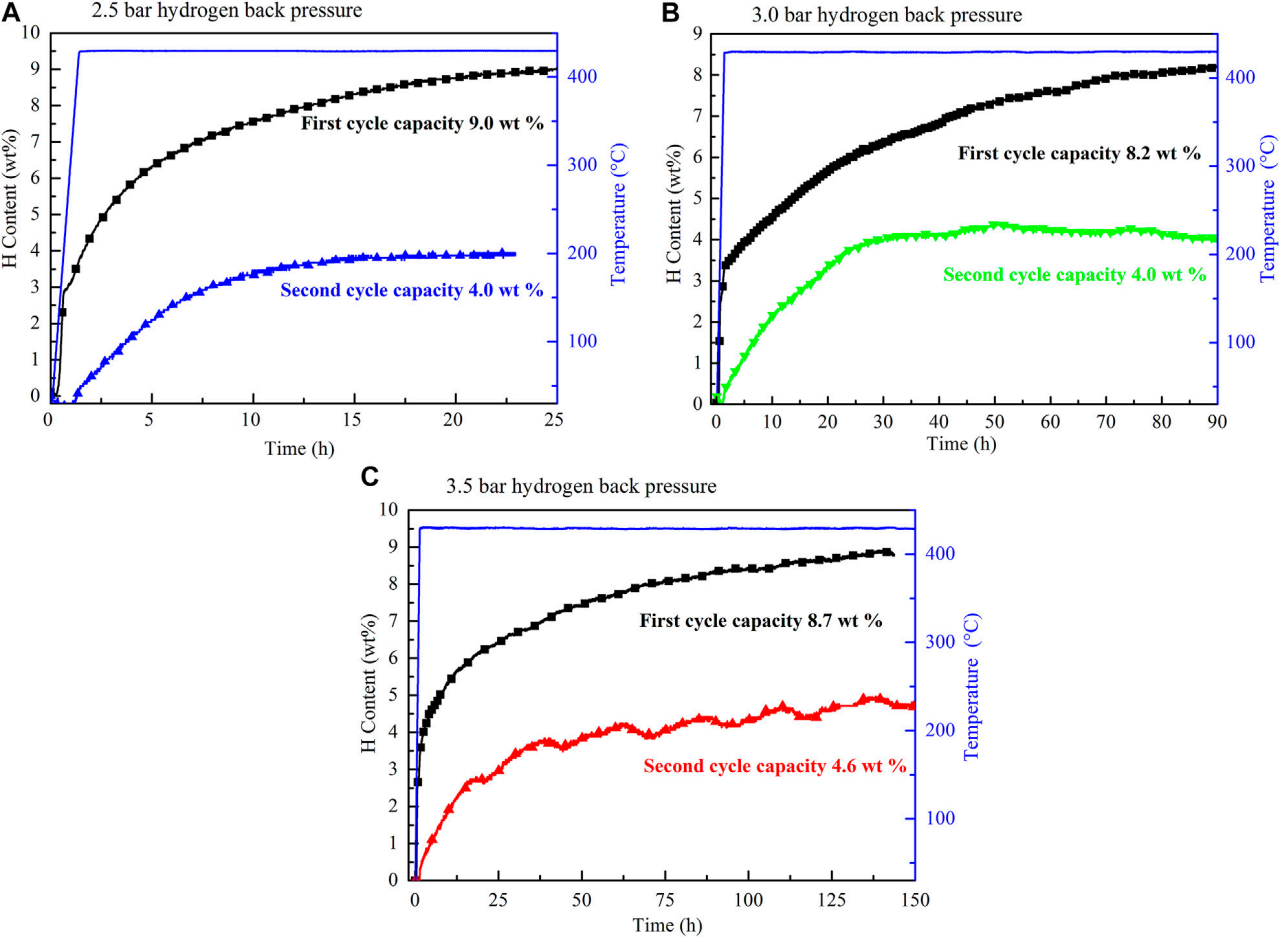
Cyclic dehydrogenation curves of the TiF_3_-added 2LiBH_4_–Li_3_AlH_6_ composites with **(A)** 2.5 bar, **(B)** 3.0 bar, and **(C)** 3.5 bar of hydrogen back pressure in the first and second dehydrogenation cycles.

In the second and third dehydrogenation cycles, the hydrogen releasing capacities were lower than 3.5 wt%, which is significantly lower than the first one. Therefore, further research is required to improve the reversible performance. In a previous work, Mao (Mao et al., 2013) reported that hydrogen back pressure could improve the cyclic performance of LiBH_4_-based composites. As mentioned earlier, TiF_3_ is a superior catalyst for improving the hydrogen storage performance of the 2LiBH4–Li_3_AlH_6_ composite. Therefore, the studies that follow focus on the influence of hydrogen back pressure on the dehydrogenation and cyclic performance of TiF_3_-added 2LiBH_4_–Li_3_AlH_6_ composites. The 2LiBH_4_–Li_3_AlH_6_ with 5 wt% TiF_3_ composite was dehydrogenated at 425°C with initial hydrogen back pressures of 2.5, 3.0, and, 3.5 bar. The dehydrogenated samples then began to absorb hydrogen at 100 bar hydrogen pressure for 10 h. The dehydrogenation curves at different hydrogen back pressures in the first and second cycles are shown in Figure 8. The dehydrogenation capacity in the first cycle at the different hydrogen back pressures was about 8.2 wt% to 9.0 wt% in the 2LiBH_4_–Li_3_AlH_6_ with 5 wt% TiF_3_ composite, which is lower than its theoretical capacity. In the second dehydrogenation cycle, the amount of hydrogen released was 4.0 wt% or 4.6 wt%, which are higher than that of 2LiBH_4_–Li_3_AlH_6_ composite (3.3 wt%). From these results, it could be concluded that a hydrogen back pressure of between 2.5 and 3.5 bar and the addition of TiF_3_ could improve the reversible performance of the 2LiBH_4_–Li_3_AlH_6_ composite to some extent. However, the dehydrogenation kinetics of the 2LiBH_4_–Li_3_AlH_6_ with TiF_3_ composite was hindered by the hydrogen back pressure. It took more than 150 h to release 8.7 wt% hydrogen with 3.5 bar hydrogen back pressure in the TiF_3_-added 2LiBH_4_–Li_3_AlH_6_ composite, compared to 1.5 h in the composite without back pressure.

**FIGURE 9:**
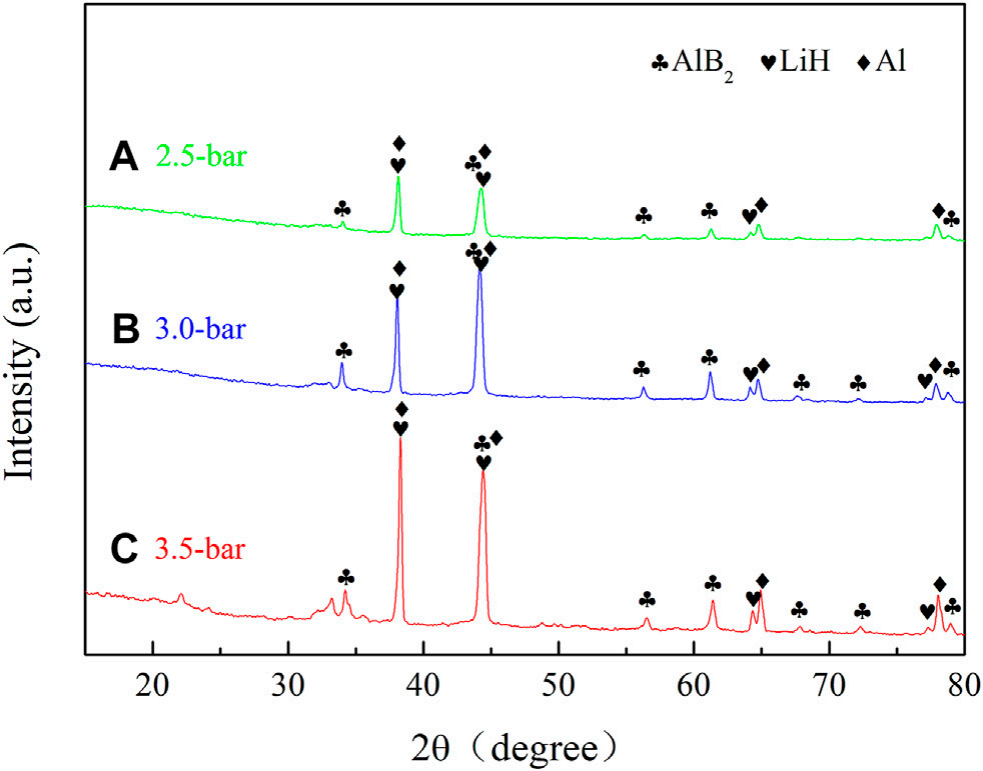
XRD patterns of the second cycle dehydrogenation products of TiF_3_-added 2LiBH_4_–Li_3_AlH_6_ composites with different back pressures: **(A)** 2.5 bar, **(B)** 3.0 bar, and **(C)** 3.5 bar.

The XRD patterns of the second-cycle dehydrogenation products are shown in Figure 9. Three phases, namely, AlB_2_, LiH, and Al, were detected in all the curves. Compared with Figure 5B, the diffraction intensity of AlB_2_ increased in Figures 9A–C, which resulted in the improved reversible performance.

**FIGURE 10:**
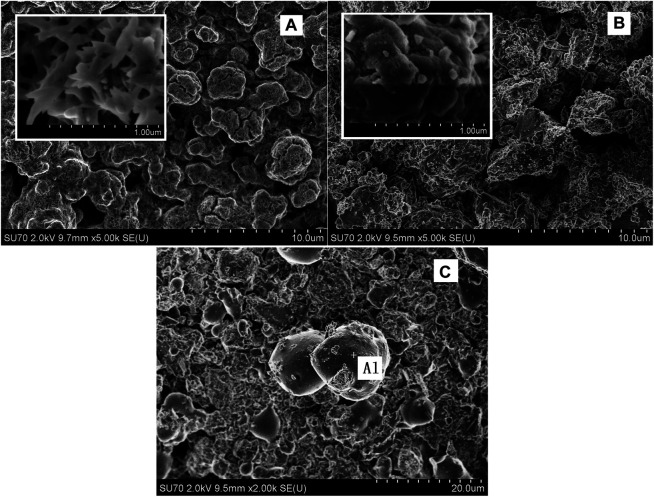
SEM images of the 2LiBH_4_–Li_3_AlH_6_ composites: **(A)** ball-milled sample, **(B)** sample after first dehydrogenation cycle, and **(C)** sample after second dehydrogenation cycle with 3 bar back pressure.

The surface topography of the 2LiBH_4_–Li_3_AlH_6_ composites under different conditions was observed by SEM. The ball-milled sample (Figure 10A) displayed a coral-like structure. At 50,000 × magnification (inset of Figure 10A), the particles had a rod-shaped appearance with a length of approximately 3 μm and a width of 1 μm. After the first cycle of dehydrogenation, the particles agglomerated into aggregates (Figure 10B) instead of rod-shaped structures. After the second cycle of dehydrogenation at the back pressure of 3 bar, some spherical particles were formed, as shown in Figure 10C. Using the energy dispersive spectrometer, these particles were found to consist of metallic aluminum. The aggregation and growth of metallic aluminum led to a poor dispersion and reduced active surface area of the dehydrogenation products, which may explain the partial reversibility of the 2LiBH_4_–Li_3_AlH_6_ composites (Meethom et al., 2020).

## Conclusion

In this study, the dehydrogenation performance and influence of back pressure on the 2LiBH_4_–Li_3_AlH_6_ with/without TiF_3_ composites were investigated. TiF_3_ enabled Li_3_AlH_6_ and LiBH_4_ to desorb hydrogen at lower temperatures. With an increase in the amount of TiF_3_, the dehydrogenation temperature decreased significantly. In the 15% TiF_3_-added composite, Li_3_AlH_6_ decomposed at 85°C and LiBH_4_ at 225°C, that is, at 105°C lower for each component compared with the undoped sample. However, when the TiF_3_ content was increased to 15 wt%, the hydrogen capacity of the composite was reduced to 7.85 wt%, which is 15% lower than that without the 2LiBH_4_–Li_3_AlH_6_ composite. In the reversibility evaluation, the hydrogen back pressures of 2.5 bar, 3.0 bar, and 3.5 bar hydrogen were adopted. The results indicate that the back pressure could enhance the reversible performance of 2LiBH_4_–Li_3_AlH_6_ composite to some extent. A reversible hydrogen capacity of 4.6 wt% was obtained with a back pressure of 3.5 bar in the TiF_3_-added 2LiBH_4_–Li_3_AlH_6_ composites. However, the kinetics of the TiF_3_-added 2LiBH4–Li3AlH_6_ composites were hindered. More than 150 h were required to complete the hydrogen release process at the hydrogen back pressure of 3.5 bar. The SEM results showed that metallic aluminum was aggregated, resulting in poor dispersion of the dehydrogenated phases and reduced active surface area. Further research is needed to improve the desorption and absorption performance of LiBH_4_.

## Data Availability

The original contributions presented in the study are included in the article/Supplementary Material, further inquiries can be directed to the corresponding author.
